# Skeletal muscle dysfunctions in pulmonary arterial hypertension: Effects of aerobic exercise training

**DOI:** 10.3389/fphys.2023.1148146

**Published:** 2023-03-23

**Authors:** Filipe Rios Drummond, Luciano Bernardes Leite, Denise Coutinho de Miranda, Lucas Rios Drummond, Victor Neiva Lavorato, Leôncio Lopes Soares, Clóvis Andrade Neves, Antônio José Natali

**Affiliations:** ^1^ Department of General Biology, Laboratory of Structural Biology, Federal University of Viçosa, Viçosa, MG, Brazil; ^2^ Department of Physical Education, Laboratory of Exercise Biology Federal University of Viçosa, Viçosa, MG, Brazil; ^3^ Department of Biological Sciences, Laboratory of Cell Signaling, Federal University of Ouro Preto, Viçosa, MG, Brazil; ^4^ Department of Physical Education, Governador Ozanam Coelho University Center (UNIFAGOC), Ubá, Brazil; ^5^ Department of Physiology and Biophysics, Laboratory of Endocrinology and Metabolism, Federal University of Minas Gerais, Belo Horizonte, MG, Brazil

**Keywords:** myopathy, pulmonary hypertension, effort, physical performance, aerobic exercise

## Abstract

Pulmonary arterial hypertension is associated with skeletal muscle myopathy and atrophy and impaired exercise tolerance. Aerobic exercise training has been recommended as a non-pharmacological therapy for deleterious effects imposed by pulmonary arterial hypertension. Aerobic physical training induces skeletal muscle adaptations *via* reduced inflammation, improved anabolic processes, decreased hypoxia and regulation of mitochondrial function. These benefits improve physical exertion tolerance and quality of life in patients with pulmonary arterial hypertension. However, the mechanisms underlying the therapeutic potential of aerobic exercise to skeletal muscle disfunctions in patients with pulmonary arterial hypertension are not well understood yet. This minireview highlights the pathways involved in skeletal muscle adaptations to aerobic exercise training in patients with pulmonary arterial hypertension.

## 1 Introduction

Aerobic exercise training (AET) is recommended for the general population, including patients with cardiovascular diseases, due to its numerous benefits already reported (Gielen et al., 2015). Studies have demonstrated that moderate-to high-intensity AET causes beneficial adaptations to the cardiovascular system of individuals affected by pulmonary arterial hypertension (PAH), as it maintains the right ventricular function (RV), which in turn preserves stroke volume and cardiac output (Nogueira-Ferreira et al., 2018; Soares et al., 2018).

The circumstances imposed by PAH impair the individual’s quality of life and develop intolerance to physical effort (Riou et al., 2020). The underlying mechanisms involved are complex as it includes central (i.e., stroke volume and cardiac output) and peripheral (i.e., blood flow, endothelial and skeletal muscle functions) adverse adaptations (Moreira-Gonçalves et al., 2015b; Soares et al., 2018). Although studies indicate benefits of AET to the cardiovascular system and consequent improvement in physical effort tolerance (Nogueira-Ferreira et al., 2018; Soares et al., 2018), there are few studies addressing the underlying mechanisms involved in skeletal muscle adaptation of individuals with PAH. Therefore, this mini-review aimed to highlight cellular and molecular pathways involved in the skeletal muscle adaptations to aerobic exercise training in patients with pulmonary arterial hypertension.

## 2 Pulmonary arterial hypertension

Pulmonary hypertension is a disorder that involves multiple clinical conditions and may aggravate most cardiovascular and respiratory diseases (Hoeper et al., 2014). Pulmonary arterial hypertension, the most common subtype of pulmonary hypertension, has an incidence of 1.1–17.6 per million adults per year and prevalence of 6.6–26.0 per million adults (Wilkins et al., 2018). Pulmonary arterial hypertension has a poor prognosis, with a mortality rate of approximately 15% in the first year after diagnosis, even with treatment (McLaughlin et al., 2009). Pulmonary arterial hypertension typically manifests in the third to fourth decade of life but can also be diagnosed in children, in whom prognosis is more severe (Ivy, 2016). Until 2019, PAH was defined clinically as mean pulmonary arterial pressure >25 mmHg at rest with normal left atrial pressure, but this definition has since been revised to mean pulmonary arterial pressure >20 mmHg, normal left atrial pressure and pulmonary vascular resistance ≥3 Wood units (Simonneau et al., 2019). Pulmonary arterial hypertension is caused by restricted blood flow in the pulmonary arterial circulation, a combination of endothelial dysfunction and increased contractility of the small pulmonary arteries. These changes are due to proliferation and remodeling of smooth endothelial muscle cells, thrombosis *in situ*, resistance to apoptosis, inflammation, and fibrosis, which results in elevated pulmonary vascular resistance (Ryan et al., 2015).

The chronic increase in pulmonary vascular resistance leads to augmented right ventricular afterload. This overload generates an adaptive response with adverse remodeling of the right ventricle. Thus, the right ventricle exhibit hypertrophy associated with elevated passive tension of the sarcomeres, collagen deposition in the extracellular matrix, fibrosis, inflammation, cellular apoptosis and, consequently, contractile dysfunction (Sandoval, 2018). These structural and functional damages lead to right ventricular failure (Taylor et al., 2020), the major cause of death in patients with PAH (De Alvaro et al., 2004).

The most commonly symptoms reported in PAH are dyspnea and fatigue, which limit the physical capacity and the quality of life (Malenfant et al., 2015). Intolerance to physical exercise is a fundamental characteristic of PAH and has traditionally been attributed to low cardiac output and respiratory dysfunction (Marra et al., 2015). However, several studies have already highlighted a wide range of abnormalities in skeletal (Bauer et al., 2007) and respiratory (Meyer et al., 2005) muscles in patients with PAH that can contribute to physical exercise limitation.

In fact, PAH decreases exercise tolerance. On the other hand, it was verified that aerobic physical exercise increases the maximum consumption of oxygen (McCullough et al., 2020), as well as increases the cross-sectional area of the skeletal muscle, which improves exercise tolerance (Moreira-Gonçalves et al., 2015a).

## 3 Pulmonary arterial hypertension and skeletal muscle

Traditionally, the loss of muscle mass leads to physical effort intolerance and is attributed to low cardiac output and consequent reduction in the availability of oxygen and other nutrients to peripheral systems (Taylor et al., 2020). It is also hypothesized that elevated inflammatory response, inhibition of anabolic pathways, hypoxemia and abnormalities in mitochondrial function contribute to explain muscle loss and dysfunction (Marra et al., 2015), despite being tested to a limited extent. Therefore, contrary to initial understanding, reduced exercise capacity is not triggered solely by cardiopulmonary compromises (Riou et al., 2020). It has been observed that the skeletal muscle impairments contribute to limiting exercise capacity, increasing sedentary behavior, and reducing activities of daily living.

Pulmonary arterial hypertension is also reported to cause an imbalance between synthesis and degradation of structural and contractile proteins in the myofibrils (Sandri, 2008). Although protein degradation occurs through multiple proteolytic systems, it has been shown in animal model that proteolysis mediated by the ubiquitin-proteasome system (UPS) is the system predominantly activated in muscle atrophy (Marra et al., 2015). In fact, high levels of atrogin-1 and muscle RING-finger protein-1 (MuRF1) were observed in the quadriceps of patients with PAH, suggesting that UPS-mediated proteolysis contributes to skeletal muscle atrophy in these patients (Batt et al., 2014).

Furthermore, it has been observed increments in the circulation of proinflammatory cytokines in individuals with PAH, which may damage muscle contractile proteins, induce proteolysis and necrosis, and lead to atrophy and decreased physical performance (Li et al., 2005; Marra et al., 2015). Such damages have already been associated with augmented levels of interleukins (IL) (IL-1β, IL-2. IL-4, IL-6, IL-8 and IL-10) and tumor necrosis factor alpha (TNF-α) in the muscle (Hassoun et al., 2009; Soon et al., 2010; Marra et al., 2015). It is suggested that the elevated circulation of pro-inflammatory markers alters the insulin receptor substrate - phosphatidylinositol 3-kinase - protein kinase B (IRS-PI3K-Akt) pathway, which is related with insulin resistance and damages to skeletal muscle (Rotter et al., 2003; De Alvaro et al., 2004; Weigert et al., 2006; Bouzakri and Zierath, 2007; Wei et al., 2008; Odegaard and Chawla, 2013).

Another proposed mechanism is the inhibition of anabolic pathways. The systemic inflammation may damage the insulin signaling, the most explored anabolic axis, in individuals with PAH (Marra et al., 2015). Thus, the IRS/PI3K/Akt axis undergoes greater inhibition and diminishes signaling for protein synthesis (Meyer et al., 2005). Moreover, it has been observed that, in heart failure, there is a decline in the levels of testosterone, which reduces the activation of the insulin-like growth factor (IGF-I) in the muscle and hence loss of muscle mass and exercise capacity limitation (Volterrani et al., 2012).

Hypoxemia has also to be considered in PAH-related myopathy. It is well established that PAH impairs microcirculation in skeletal muscle (Marra et al., 2015). This modification dwindles the availability of oxygen in the musculature, hence limiting the aerobic and augmenting the anaerobic metabolism. For instance, impairment of oxidative enzyme activity, elevated activity of glycolytic enzymes, hypercapnia and metabolic acidosis have been observed in PAH (Tran et al., 2018).

Beside these mechanisms, abnormalities in mitochondrial function can contribute to the development of PAH-related myopathy. The study by Kue et al. (2017) reported that the soleus muscle of animals with PAH showed a reduction in the expression of the coactivator-1 alpha of the receptor activated by gamma peroxisome proliferators (PGC1α) and mitochondrial DNA (mtDNA), without alteration in OPA1, thus, impairing mitochondrial biogenesis. Additionally, McCullough et al. (McCullough et al., 2020) demonstrated that PAH induced by Sugen/Hypoxia promoted negative changes in the expression of electron transport chain supercomplexes in the gastrocnemius of rats. Mitochondrial supercomplexes are dynamic sets of individual complexes in the electron transport chain that can lead to more efficient respiration (Genova and Lenaz, 2014).

Although the underlying mechanisms of skeletal muscle dysfunction in PAH is still not completely known, the effect of this pathology on skeletal muscle and on the quality of life of patients is undeniable. Therefore, studies seeking to clarify the mechanisms involved, as well as therapies capable of reversing or mitigating the damage to the muscular system are of interest and of clinical relevance.

## 4 Pulmonary arterial hypertension and physical exercise

The current pharmacological therapies for the treatment of PAH have positive effects, however such drugs are not always available and some patients are non-responders (Sandoval, 2018). Consequently, the search for efficient therapies are of interest to treat this disease and improve the quality of life of patients.

Aerobic exercise training has been recommended as a valuable non-pharmacological therapeutic tool for the treatment of several chronic diseases, such as pulmonary, cardiovascular, and musculoskeletal disorders (Pedersen and Saltin, 2015). Current reviews (Nogueira-Ferreira et al., 2018; Soares et al., 2018) presented evidences in humans and in animal models that AET triggers positive adaptations in individuals with PAH, such as enhanced functional and exercise capacity, ventilatory efficiency, global cardiac function, arterial and myocardial elasticity, antioxidative and anti-inflammatory defense, and cardiopulmonary remodeling (Souza-Rabbo et al., 2008; De Man et al., 2009; Handoko et al., 2009; Shoemaker et al., 2009; Moreira-Gonçalves et al., 2015a; Buys et al., 2015; Natali et al., 2015; Brown et al., 2017; Soares et al., 2019; De Jesus Silva et al., 2021).

Non-etheless, according to Nogueira-Ferreira et al. (2018), there are few clinical and experimental studies on the effects of AET on the skeletal muscle in PAH, which limits the understanding of the underlying mechanisms for such effects. As mentioned above reported, it is conceivable that damages caused to the skeletal muscle by PAH due to increases in circulating proinflammatory cytokines (Marra et al., 2015), though, we found no study showing the effects of AET on the inflammatory state of skeletal muscle in patients with PAH. On the other hand, some studies demonstrated the ability to AET in inducing the synthesis and secretion of myokines such as IL-6, IL-8, IL-10 and IL-15 (Benatti and Pedersen, 2015; Hoffmann and Weigert, 2017). It is suggested that the main function of myokines is to protect the functionality of the musculature against the altered inflammatory state (Hoffmann and Weigert, 2017). For instance, Benatti and Pedersen. (2015) pointed out that myokines (e.g., IL-6) can induce an acute anti-inflammatory response after each exercise session. According to Steensberg et al. (2003) after IL-6 is released into the bloodstream, it induces a subsequent increment in the production of the IL-1 agonist receptor (IL-1ra) and IL-10 by mononuclear leukocytes in the blood, hence generating an anti-inflammatory. In addition to acute exercise, long-term aerobic and resistance training also augment the synthesis other myokines such as IL-15, which may mitigate some cardiovascular risk factors and thus having indirect anti-inflammatory effects (Pedersen and Febbraio, 2012). Therefore, it is conceivable that physical training has important anti-inflammatory effects on skeletal muscle that contribute to greater tolerance to physical exertion, since the deposition of fibrous tissue can be reduced, as well as the loss of muscle mass.

Experimental studies indicate that AET can prevent or even attenuate the inhibition of anabolic hormone pathways. For example, Gonçalves et al. (Gonçalves et al., 2012) showed that AET for 4 weeks before monocrotaline-induced PAH, followed by 4 weeks of sedentary behavior, prevented the reduction of myosin heavy chain I (MHC-I) isoform expression in gastrocnemius of rats, in addition to preventing the reduction of the cross-sectional area of muscle fibers. AET before PAH also increased phosphorylated forms of Akt and the mammalian target of rapamycin (mTOR), involved in the protein synthesis pathway, while restricting atrogin-1 expression. Atrogin-1 is a cardiac and skeletal muscle specific F-box protein that has anti-hypertrophic capacity and is a crucial player in skeletal muscle atrophy events (Gomes et al., 2001). Furthermore, Moreira-Gonçalves et al. (2015b) reported that 4 weeks of AET performed after MCT application in rats resulted in increased weight and gastrocnemius cross-sectional area. The aforementioned results show that AET is able to increase skeletal muscle hypertrophy, collaborating to increase strength and, consequently, leading to greater physical independence.

Regarding hypoxemia, a clinical study on patients with idiopathic PAH, demonstrated that 12 weeks of exercise training (i.e., cycling and quadriceps strength and endurance) increased the muscular resistance of these individuals (De Man et al., 2009). This improvement was explained by the 30% increase in the number of blood capillaries and the 39% augment in the absorbance of the oxidative enzyme succinate dehydrogenase in the quadriceps. In addition to these findings, Vieira et al. (Vieira et al., 2020) showed that rats treated with monocrotaline and trained on a treadmill increased the cross-sectional area of type IIX muscle fibers. These results suggest that physical training induces mechanisms that could attenuate muscle hypoxemia caused by PAH, leading to improved muscle oxygenation and lower production of reactive oxygen species. However, the mechanisms still need to be better explored.

Concerning muscle metabolism, McCullough et al. (McCullough et al., 2020), reported recently that AET after experimental PAH induced by Sugen/Hypoxia increased mitochondrial supercomplexes in the electron transport chain in the oxidative portion of the gastrocnemius in rats. Thus, it is suggested that AET enhances both function and structure of mitochondria. In this way, the skeletal muscle would have better use in the generation of energy, which leads to the improvement of movement mechanics.

Other conditions such as chronic obstructive pulmonary disease (COPD) and heart failure, which may result from PAH, induce the aforementioned skeletal muscle impairments, with increases in atrogin-1 (Bodine and Baehr, 2014; Yoshida and Delafontaine, 2015), pro-inflammatory cytokines (Barnes, 2016), reduced capillarization and increased number of type II muscle fibers (Jobin et al., 1998) and mitochondrial dysfunction (Mathur et al., 2014; Kinugawa et al., 2015). As with PAH, physical exercise attenuates the deleterious effects of COPD and heart failure (Cunha et al., 2012; Zhang et al., 2019; Broxterman et al., 2021).

**FIGURE 1:**
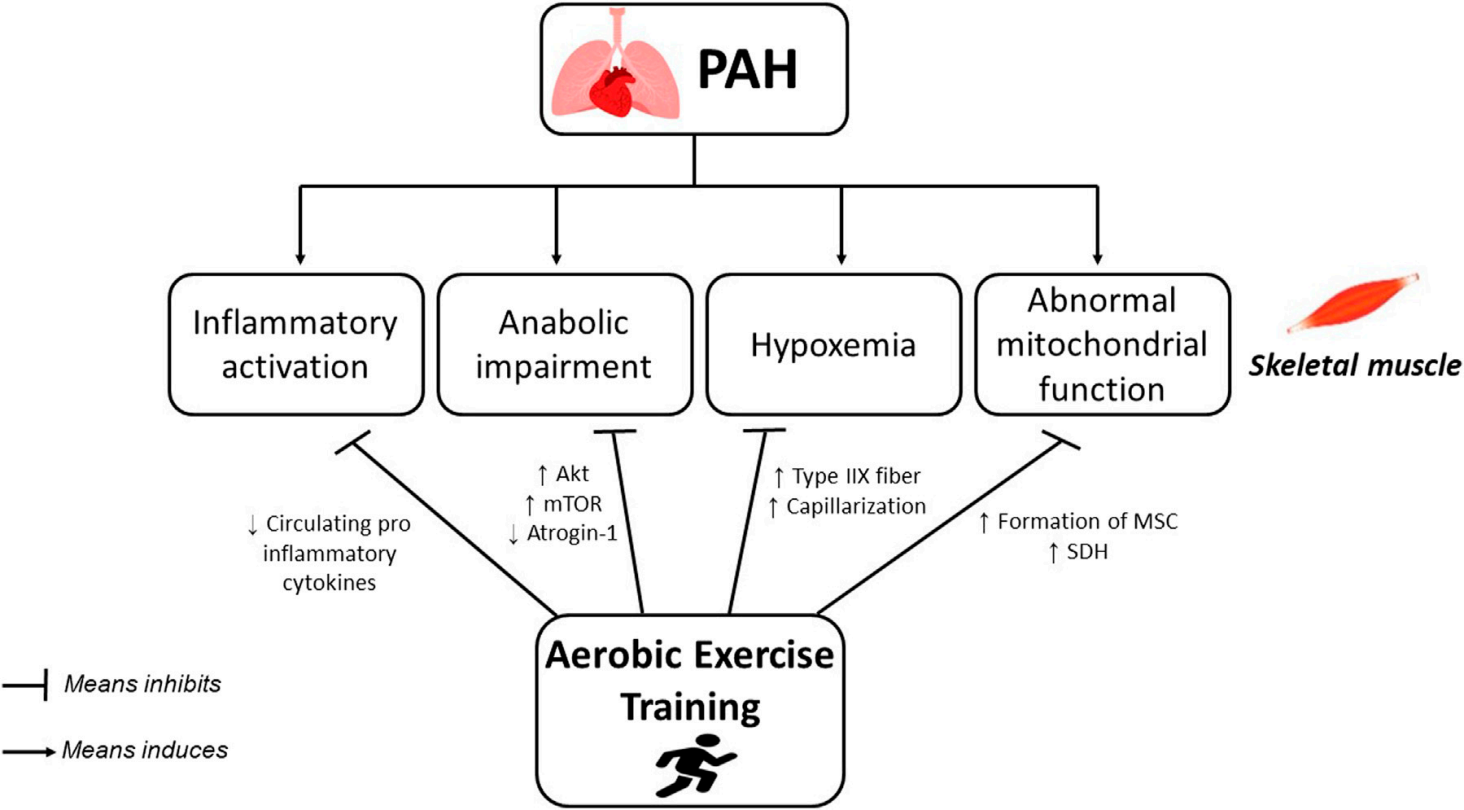
Potential mechanisms underlying the effects of aerobic exercise training on skeletal muscle atrophy and weakness in PAH.

Finally, Figure 1 resumes the proposed mechanisms reported in the literature that helps to the skeletal muscle structural and functional changes in patients with PAH undergoing AET.

## 5 Perspectives

In this mini-review, we highlighted the main cellular and molecular adaptations reported in the literature that could explain in part the skeletal muscle structural and functional changes in patients with PAH undergoing AET. Despite the lack of studies addressing the skeletal muscle adaptations AET in these patients, it is observed that AET can provide protection and reduce muscle atrophy, increased muscle capillarization and improvement in mitochondrial function. Nevertheless, studies on the effects of physical exercise on skeletal muscle structural (i.e., morphology and muscle mass), cellular (i.e., changes in the type of muscle fiber and connective tissue) and molecular (i.e., alterations in contractile units of muscle fibers; muscle inflammatory state; stress oxidative; signaling pathways for cell survival and death; biogenesis and mitochondrial metabolism; and epigenetic analyzes, specifically of miRNAs linked to contractile mechanics) adaptations in patients with PAH are of interest for the understanding the mechanisms that involve the adaptations of the skeletal muscle AET and thus obtain important tools to mitigate the damage imposed by the disease.
